# Fucosyltransferase 1 mediates angiogenesis, cell adhesion and rheumatoid arthritis synovial tissue fibroblast proliferation

**DOI:** 10.1186/ar4456

**Published:** 2014-01-28

**Authors:** Takeo Isozaki, Jeffrey H Ruth, Mohammad A Amin, Phillip L Campbell, Pei-Suen Tsou, Christine M Ha, G Kenneth Haines, Gautam Edhayan, Alisa E Koch

**Affiliations:** 1 Department of Internal Medicine, University of Michigan, Ann Arbor, MI, USA; 2 Currently Department of Internal Medicine, Showa University School of Medicine, Tokyo, Japan; 3 Department of Pathology, Yale University, New Haven, CT, USA; 4 VA Medical Service, Department of Veterans Affairs Medical Center, Ann Arbor, MI 48108, USA

## Abstract

**Introduction:**

We previously reported that sialyl Lewis^y^, synthesized by fucosyltransferases, is involved in angiogenesis. Fucosyltransferase 1 (fut1) is an α(1,2)-fucosyltransferase responsible for synthesis of the H blood group and Lewis^y^ antigens. However, the angiogenic involvement of fut 1 in the pathogenesis of rheumatoid arthritis synovial tissue (RA ST) has not been clearly defined.

**Methods:**

Assay of α(1,2)-linked fucosylated proteins in RA was performed by enzyme-linked lectin assay. Fut1 expression was determined in RA ST samples by immunohistological staining. We performed angiogenic Matrigel assays using a co-culture system of human dermal microvascular endothelial cells (HMVECs) and fut1 small interfering RNA (siRNA) transfected RA synovial fibroblasts. To determine if fut1 played a role in leukocyte retention and cell proliferation in the RA synovium, myeloid THP-1 cell adhesion assays and fut1 siRNA transfected RA synovial fibroblast proliferation assays were performed.

**Results:**

Total α(1,2)-linked fucosylated proteins in RA ST were significantly higher compared to normal (NL) ST. Fut1 expression on RA ST lining cells positively correlated with ST inflammation. HMVECs from a co-culture system with fut1 siRNA transfected RA synovial fibroblasts exhibited decreased endothelial cell tube formation compared to control siRNA transfected RA synovial fibroblasts. Fut1 siRNA also inhibited myeloid THP-1 adhesion to RA synovial fibroblasts and RA synovial fibroblast proliferation.

**Conclusions:**

These data show that α(1,2)-linked fucosylated proteins are upregulated in RA ST compared to NL ST. We also show that fut1 in RA synovial fibroblasts is important in angiogenesis, leukocyte-synovial fibroblast adhesion, and synovial fibroblast proliferation, all key processes in the pathogenesis of RA.

## Introduction

The pathogenesis of rheumatoid arthritis (RA) is characterized by the infiltration of inflammatory cells into the pannus, followed by tissue destruction. The RA synovium contains elevated levels of cytokines and inflammatory cells such as lymphocytes and monocytes [1,2]. Chemokines and other inflammatory mediators drive the pathogenesis of RA and regulated production of proinflammatory cytokines is important for the orchestration of the inflammatory response [3-5]. Current therapies are designed to block cytokines such as TNF-α or IL-6 [6,7]. However, despite the success of blocking these cytokines, not all RA patients respond adequately to anti-TNF-α or anti-IL-6 therapy.

Angiogenesis is a highly regulated process that results in the formation of new vessels. It is important in vasculoproliferative states such as wound repair and chronic inflammation, as seen in RA [8,9]. The angiogenic process is important in the progression of RA and may prove to be a promising therapeutic target [10]. Cellular adhesion molecules expressed on endothelial cells (ECs) are involved in leukocyte extravasation into the synovium leading to perpetuation of RA synovial inflammation [11].

Glycosylation is one of the most common posttranslational modification reactions, and many proteins in eukaryotes are glycosylated [12]. Most of these are *N*-linked and/or *O*-linked glycan chains that are synthesized posttranslationally in the endoplasmic reticulum and the Golgi apparatus by various glycosyltransferases [13]. Fucosylated glycans are synthesized by fucosyltransferases (futs). Thirteen fut genes have been identified in the human genome [14]. Fucosyltransferase 1 (fut1) and fut2 are α(1,2)-fucosyltransferases responsible for synthesis of the H blood group antigen and related structures [15,16]. Fut1 is overexpressed in some cancers such as colon and pancreas [17,18]. These reports indicate that α(1,2)-linked fucose synthesized by futs are important for tumor growth. In regards to arthritis, mRNA levels of fut7 are upregulated in synovial fluid (SF) compared to peripheral blood T cells in patients with juvenile idiopathic arthritis [19].

We have shown previously that the soluble form of E-selectin mediates angiogenesis via its endothelial ligand sialyl Lewis^x^[20]. We have also shown that Lewis^y^-6/H/5-2 (Le^y^/H), synthesized by fut1, and its glucose analog, 2-fucosyllactose (H-2 g) mediates angiogenesis and inflammatory cell adhesion [21,22]. However, a direct role for fut1 in RA has not been demonstrated. In this study, we found that α(1,2)-linked fucosylated proteins were expressed in RA synovium. Hence, we show that α(1,2)-linked fucosylated proteins are upregulated in RA synovial tissue (ST) and that fut1 in RA synovial fibroblasts is important in EC tube formation, leukocyte-synovial fibroblast adhesion, and synovial fibroblast proliferation, all critical aspects of inflammation in the RA joint synovium.

## Methods

### Patients

RA and osteoarthritis (OA) ST were obtained from patients undergoing arthroplasty or synovectomy. Normal (NL) ST samples were obtained from a National Disease Research Interchange and Cooperative Human Tissue Network. NL skin biopsies were obtained from the University of Michigan Tissue Procurement Service. For all human specimens used in this study, we obtained written informed consent with approval from the University of Michigan Institutional Review Board.

### Homogenate preparation

ST homogenates were prepared in anti-protease buffer as we have done previously [23]. Briefly, RA, OA and NL STs were homogenized in 5 ml of a 1% protease inhibitor cocktail (Pierce, Rockford, IL, USA) in PBS. Samples were centrifuged at 900 g for 10 minutes, and filtered through a 1.2-μm pore size Sterile Acrodisk, and frozen at -80°C until thawed for assay. The total protein concentration of each lysate was determined using a bicinchoninic acid assay (Pierce).

### Cell culture

Fresh STs were minced and digested in tissue enzyme digestion solution as described previously [24]. The synovial fibroblasts were maintained in Roswell Park Memorial Institute (RPMI) 1640 medium supplemented with 10% FBS. Cells were seeded in 6-well plates (BD Biosciences, Bedford, MA, USA) at a density of 1 × 10^5^ cells per well, and were maintained in complete medium. After overnight serum starvation, cells were treated with 25 ng/ml TNF-α (R&D Systems, Minneapolis, MN, USA) for 24 hours. Cell-conditioned medium and cell lysates were collected. We used fibroblasts from three NL knees; three knees and one hip from OA patients; and six knees from RA patients.

Human dermal microvascular endothelial cells (HMVECs) were purified from digested skin biopsies using mouse anti-human CD31 MicroBeads (Miltenyi Biotec, Cambridge, MA, USA), according to the manufacturer’s protocol. Cells were cultured in EC basal medium (Lonza, Walkersville, MD, USA) with growth factors. In order to confirm EC purity, we used antibodies to EC markers von Willebrand factor and CD31 and immunohistochemistry. THP-1 cells (human acute monocytic leukemia cell line) were purchased from the American Type Culture Collection (Manassas, VA, USA). THP-1 cells were cultured in RPMI supplemented with 10% FBS.

### Enzyme-linked lectin assay (ELLA)

For RA, OA and NL ST homogenate analysis, we measured the total protein content of each of our samples as done previously [25]. We next used Ulex Europeaus Agglutinin 1 lectin (UEA-1) to measure α(1,2)-linked fucosylated proteins in the same samples. UEA-1 binds specifically to α(1,2)-fucose, the terminal sugar of blood group antigens H and Lewis^y^. 2′fucosyllactose-bovine serum albumin (2′FL-BSA), 50 to 0.78 ng/ml (V-labs Inc, Covington, LA, USA) was used as a standard for measurement of total fucosyated proteins. Because 2′FL-BSA is linked to α(1,2)-fucose on BSA, and BSA is a well known standard for measuring proteins, we used 2′FL-BSA as a metric for quantifying total α(1,2)-linked fucosylated proteins in our samples. Results are presented as the ratio of the total fucosylated proteins in each of the ST homogenate samples using fucosylated BSA as standard (in ng of 2′FL-BSA), normalized to the total protein content of the same sample (in mg) as measured using nonfucosylated BSA as a standard (Pierce).

The ELLA was performed by adding samples and standards to 96-well plates with an overnight incubation at 4°C. The next day, plates were washed (PBS + 0.05% Tween), and blocked with Synblock (ImmunoChemistry Tech, Bloomington, MN, USA) for 2 hours. UEA-1, 2 μg/ml (Vector laboratories Inc, Burlingame, CA, USA) was added for 90 minutes followed by 10 μg/ml biotinylated goat anti-UEA-1 antibody (Vector laboratories Inc) for 60 minutes, then streptavidin-horseradish peroxidase (HRP) for 30 minutes. Tetramethylbenzine substrate (TMB) was used as a color development reagent and the plate was read at 450 nm following addition of 1 N H_2_SO_4_ on a BioTek Synergy plate reader (Winooski, VT, USA). Total fucosylated proteins from synovial fibroblast conditioned medium and cell lysates were measured by ELLA using 2′FL-BSA as a standard curve.

### Immunofluorescence

Immunofluorescence staining on RA ST fibroblasts was performed as previously described [25]. To determine if α(1,2)-linked proteins were expressed on RA ST synovial fibroblasts, mouse anti-human collagen-1 (Abcam, Cambridge, MA, USA) and goat anti-UEA-1 (Vector laboratories Inc.) were used. RA ST slides were fixed with cold acetone for 20 minutes. Then slides were blocked with 20% FBS and 5% donkey serum for 1 hour at 37°C, and incubated with UEA-1 (2 μg/ml, Vector laboratories Inc.) for 1 hour at 37°C. Goat anti-UEA-1 and rabbit anti-human collagen-1 were used as primary antibodies. Fluorescent conjugated donkey anti-goat (for UEA-1) and anti-mouse (for collagen-1) secondary antibodies were purchased from Life Technologies (Carlsbad, CA, USA). For nuclear staining, 4′, 6-diamidino-2-phenylindole (DAPI) was used. Images were taken at 100× magnification. Anti-collagen-1-positive fibroblasts were shown by fluorescent red staining, and fucosylation was shown by fluorescent green. Yellow cells were a result of merging the green and red fields.

Dual immunofluorescence staining was done on RA ST sections embedded in optimal cutting temperature (OCT) medium and cryosectioned. The slides were fixed and blocked in 5% goat serum. Rabbit anti-human fut1 (Thermo Scientific, Waltham, MA), mouse anti-human CD68 macrophage marker (BD Biosciences, San Jose, CA), and mouse anti-human Cadherin-11 (fibroblast marker; R&D Systems) were used as primary antibodies at a concentration of 10 μg/ml and incubated for 1 hour at 37°C. The slides were washed with PBS and goat anti-rabbit fluorescein isothiocyanate (FITC)-conjugated and goat anti-mouse rhodamine-conjugated antibodies were used as secondary antibodies and were incubated for 1 hour at 37°C. The slides were again washed in PBS and 4′, 6-diamidino-2-phenylindole (DAPI) staining was used at 1:5,000 concentration. The slides were mounted and visualized under tetramethylrhodamine (TRITC), FITC, and DAPI wavelengths.

### Immunohistologic analysis

We performed immunoperoxidase staining on cryosections from NL, OA, and RA ST as described previously [25]. ST samples were blocked with 20% FBS and 5% goat serum in PBS, and incubated with mouse anti-human fut1 (10 μg/ml, Thermo Scientific) or purified nonspecific mouse IgG. ST samples were washed with PBS, and a 1:200 dilution of biotinylated goat anti-mouse antibody was added. After washing, antibody binding was detected using a Vectastain ABC Elite peroxidase system (Vector laboratories Inc.) and chromogen 3,3′-diaminobenzidine (DAB) (Kirkegaard & Perry Laboratories Inc., Gaithersburg, MD, USA). ST samples were counterstained with Harris hematoxylin. Staining was evaluated by a pathologist blinded to the experimental conditions. Each of the ST components was graded for immunostaining and scored 0% to 100%, in which 0% indicates no staining and 100% indicates that all cells were immunoreactive [25].

### Transfection of RA synovial fibroblasts with fut1 small interfering RNA (siRNA)

RA synovial fibroblasts were seeded in 6-well plates at a density of 1 × 10^5^ cells per well. Cells were maintained in complete medium up to 70% confluency. siRNA (50 nM) against fut1 or control siRNA and transfection regent (Mirus, Madison, WI, USA) was mixed with TransIT-TKO transfection reagent according to manufacturer’s instructions and overlaid on the cells. Cells were incubated with the siRNA/TransIT-TKO for 24 hours at 37°C. Control and fut1 siRNA were purchased from Santa Cruz Biotechnology. To determine the transfection efficiency of cells, a fluorescein-conjugated nonsilencing siRNA (Santa Cruz Biotechnology) was transfected into cells with TransIT-TKO. Transfected cells were counted by fluorescence microscopy, and total cells were counted by bright field microscopy. The transfection efficiency was calculated as the percent of fluorescein-positive cells divided by the number of bright field cells. The percent knockdown of fut1 expression was confirmed using quantitative polymerase chain reaction (qPCR) and western blotting.

### Cell lysis and western blotting

Cell lysis and western blotting were performed as previously described [25]. RA synovial fibroblasts were transfected with fut1 siRNA or control siRNA. Cells were seeded in 6-well plates at a density of 1 × 10^5^ cells per well. After overnight serum starvation, cells were stimulated with TNF-α (25 ng/ml). Membranes were probed with rabbit anti-human fut1 antibody (Epitomics Inc., Burlingame, CA, USA). The immunoblots were stripped and reprobed with rabbit anti-β-actin to verify equal loading. For cell signaling experiments, antibodies against phosphorylated and total JNK, NFκB, P38, and Erk1/2 (Cell Signaling Technology, Danvers, MA, USA) were used.

### Co-culturing HMVECs and RA synovial fibroblasts in the EC tube formation assay

In order to confirm the effect of fut1 on HMVEC tube formation, a facet of angiogenesis, we co-cultured HMVECs and RA synovial fibroblasts using a Costar transwell system (Corning Inc., Lowell, MA, USA). RA synovial fibroblasts were first transfected with either control or fut1 siRNA as described above and were plated in the top inserts of the transwell system. HMVECs were grown on the bottom wells. HMVECs and RA synovial fibroblasts were co-cultured with serum-free endothelial basal medium (EBM) for 24 hours. HMVECs were collected from the co-culture plates, and subsequently plated on Matrigel (BD Biosciences) with the co-cultured conditioned media for 6 hours at 37°C. Tubes formed by HMVECs were counted by a blinded observer [26].

### RNA extraction and qPCR of RA synovial fibroblasts

RNA extraction and qPCR were performed as previously described [27]. Fut1, monocyte chemoattractant protein 1 (MCP-1)/CCL2, epithelial-derived neutrophil-activating peptide 78 (ENA-78)/CXCL5, vascular endothelial growth factor (VEGF) and β-actin primer pairs were purchased from Integrated DNA Technologies (Coralville, IA, USA). The following primers were used; fut1 forward 5′-GTGCCCGTATCCAGAGTGAT-3′; reverse 5′-AGGACCCAGGGGAGAGTAAA-3′; MCP-1/CCL2 forward 5′-TCCAGCATGAAAGTCTCTGC-3′; reverse 5′-TGGAATCCTGAACCCACTTC-3′; ENA-78/CXCL5 forward 5′-GAGAGCTGCGTTGCGTTTG-3′; reverse 5′-TTTCCTTGTTTCCACCGTCCA-3′; VEGF forward 5′-ATGAACTTTCTGCTGTCTTGGGT-3′; reverse 5′-TGGCCTTGGTGAGGTTTGATCC-3′; β-actin forward 5′-GCTAGGCAGCTCGTAGCTCT-3′; reverse 5′-GCCATGTACGTTGCTATCCA-3′. All samples were run in duplicate and analyzed using Applied Biosystems software (Life Technologies).

### ELISA for MCP-1/CCL2, ENA-78/CXCL5, and VEGF

ELISA was performed in a manner described previously [28]. Fut1 siRNA, control siRNA or nontreated RA synovial fibroblasts were stimulated with TNF-α (25 ng/ml) for 24 hours, and cell supernatants were collected. Levels of MCP-1/CCL2, ENA-78/CXCL5, and VEGF were measured.

### *In vitro* cell adhesion assay

Adhesion of THP-1 cells to nontreated, control siRNA or fut1 siRNA treated RA synovial fibroblasts grown to confluence in 96-well plates was examined [25]. RA synovial fibroblasts were serum-starved overnight. The next day, cells were treated with TNF-α (25 ng/ml) for 24 hours. THP-1 cells were collected and labeled with 5 μM Calcein AM fluorescent dye (Life Technologies) for 30 minutes. After washing twice, 1 × 10^5^ THP-1 cells were added to each well and incubated for 30 minutes at room temperature. Nonadherent cells were washed off and fluorescence was measured using a Synergy HT fluorescence plate reader (BioTek Instruments, Winooski, VT).

### Cell surface ELISA for adhesion molecule expression

Nontreated, control siRNA-transfected, or fut1 siRNA-transfected RA synovial fibroblasts (1 × 10^5^/well) were seeded in 96-well plates. Confluent RA synovial fibroblasts were serum-starved overnight prior to stimulation with TNF-α (25 ng/ml) for 24 hours. Cells were fixed with 3.7% formalin in PBS, and cell surface ELISA was performed as previously described [29]. Mouse anti-human antibodies specific for intercellular adhesion molecule 1 (ICAM-1), 10 μg/ml, (R&D Systems) or vascular cell adhesion molecule 1 (VCAM-1) were used, and the plates were read with an ELISA reader at 450 nm.

### Cell proliferation assay

Control or fut1 siRNA-transfected RA synovial fibroblasts were seeded in 96-well plates at 5 × 10^4^ cells/ml. Cells were serum-starved overnight then treated with 10 μg/ml lipopolysaccharide (LPS) from *Escherichium coli* 0111 (Sigma-Aldrich) for 4 and 24 hours. Each treatment group experiment was performed in four replicate wells. DNA was measured using a CyQuant cell proliferation assay kit (Life Technologies) following the manufacturer’s instructions. For the assay, cells were lysed and total cellular nucleic acid was measured using fluorescence at 520 nm emission after excitation at 480 nm.

### Statistical analysis

All data were analyzed using parametric tests, namely the Student’s *t*-test assuming equal variances. Data are reported as the mean ± standard error of the mean (SEM). *P*-values less than 0.05 were considered statistically significant. All error bars represent the SEM and n represents the number of independent experiments. Of note, all data represented in the manuscript that were significant using a parametric test, were similarly significant using the one-tailed Mann Whitney test.

## Results

### α(1,2)-linked fucosylated proteins are expressed in RA ST

**Figure 1:**
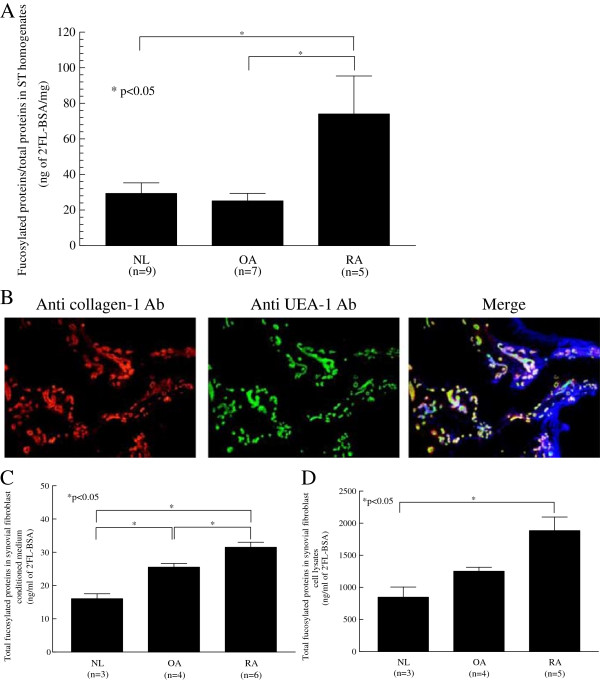
**α(1,2)-linked fucosylated proteins are expressed in rheumatoid arthritis (RA). (A)** RA synovial tissue (ST) homogenates contained more α(1,2)-linked fucosylated proteins than did osteoarthritis (OA) or normal (NL) STs (normalized to total protein concentration). Results are expressed as a ratio of the amount of fucosylated BSA/total proteins in the ST homogenates using BSA as a standard. **(B)** Photographs of RA ST. The far left panel shows staining with mouse anti-collagen 1. The middle panel shows staining with Ulex Europeaus Agglutinin 1 lectin (UEA-1) and goat anti-UEA-1. The right panel shows merging of the left panel and middle panel. Yellow indicates α(1,2)-linked fucosylated proteins associated with RA ST fibroblasts and the blue indicates DAPI staining of the tissue (original magnification 100×). **(C)** α(1,2)-linked fucosylated proteins in RA synovial fibroblast-conditioned medium were significantly higher than in OA and NL synovial fibroblast-conditioned medium. **(D)** α(1,2)-linked fucosylated proteins in RA synovial fibroblast cell lysates were significantly higher than in NL synovial fibroblast cultures: 2′fucosyllactose-bovine serum albumin (2′FL-BSA) was used as a standard.

We hypothesized that α(1,2)-linked fucosylated proteins are important in RA inflammation and play a role in angiogenesis, cell adhesion, and cell proliferation. To test this hypothesis, we determined whether α(1,2)-linked fucosylated proteins in RA synovium were higher than in other synovia. We found that RA ST homogenates contained significantly more fucosylated proteins than did either OA or NL STs (Figure 1A).

In order to determine the location of α(1,2)-linked fucosylated proteins expressed within the ST samples, we performed immunofluorescence staining. We found that α(1,2)-linked fucosylated proteins were expressed on RA ST fibroblasts (Figure 1B). In addition, we found that α(1,2)-linked fucosylated proteins in RA synovial fibroblast-conditioned medium and cell lysates (30 ± 2 ng/ml of 2′FL-BSA and 1485 ± 204 ng/ml of 2′FL-BSA, respectively) were significantly higher compared to NL synovial fibroblasts (16 ± 1 ng/ml of 2′FL-BSA and 847 ± 159 ng/ml of 2′FL-BSA, respectively (Figure 1C and D).

### Fut1 is expressed on RA ST lining cells, fibroblasts and macrophages

**Figure 2:**
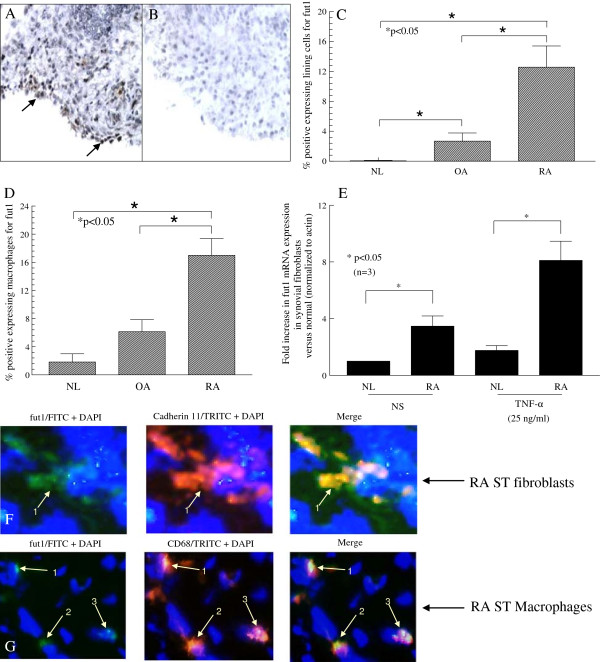
**Immunohistologic analysis of fucosyltransferase 1 (fut1) expression. (A** and **B)** Photomicrographs of ST samples from patients with rheumatoid arthritis (RA). Cryosections were stained with anti-fut1 **(A)** or control IgG **(B)**. Original magnification is 400×. Arrows indicate fut1 expression. RA synovial tissues (STs) contain a greater percentage of fut1 lining cells **(C)** compared to OA and NL ST. A significantly elevated percentage of macrophage staining on RA compared to OA or NL STs was also found **(D)**. Expression of fut1 mRNA in TNF-α stimulated or nonstimulated RA synovial fibroblasts was significantly elevated compared to TNF-α stimulated or nonstimulated NL synovial fibroblasts **(E)**. Means are presented with standard error. *P <0.05 was significant. NS = nonstimulated. (n = number of RA patients or patient ST fibroblasts). **(F)** Left panel, fut1 straining in RA ST (green); middle panel, cadherin-11 staining in RA ST (red); right panel, merge of the previous two figures. The arrow indicates fut1 and cadherin-11-positive cells respectively (yellow), indicating that fut1 is expressed on fibroblasts in RA ST. **(G)** Left panel, fut1 straining in RA ST (green); middle panel, CD68 staining in RA ST (red); right panel, merge of the previous two panels. The arrows indicate fut1 on CD68-positive cells, validating that fut1 is expressed on macrophages in RA ST (yellow). The blue background is 4′,6-diamidino-2-phenylindole (DAPI) staining. IgG control staining was performed and showed no fluorescence staining (data not shown; all figures are 400× magnification).

We determined whether fut1 was present in STs. Figures 2A and B show a photomicrograph using anti-fut1 (Figure 2A) or non-specific IgG (Figure 2B). The photomicrograph shown is from an RA ST showing 60%-positive lining cell staining, with the lining layer being composed of macrophages and fibroblasts. RA STs contained a greater percentage of fut1 positive lining cells than did OA or NL STs; mean number of lining cells ± SEM; RA ST (n = 26) 13 ± 3%; OA ST (n = 22) 3 ± 1% and NL ST (n = 18) 0 ± 0%, *P* <0.05 between RA and OA ST; RA and NL ST (Figure 2C). We found ECs expressing fut1 in RA, OA and NL ST, although the percentage of positive fut1 staining on ECs was relatively low for all groups (<2%). We also found that RA STs contained a greater percentage of fut1-positive synovial macrophages than did OA or NL STs; mean number of synovial macrophages ± SEM; RA ST (n = 26) 17 ± 2%; OA ST (n = 22) 6 ± 2% and NL ST (n = 18) 2 ± 1%, *P* <0.05 between RA and OA ST; RA and NL ST (Figure 2D).

In addition, to determine whether fut1 was expressed in RA synovial fibroblasts, qPCR was performed. We found that expression of fut1 mRNA in nonstimulated RA synovial fibroblasts was significantly higher than in nonstimulated NL synovial fibroblasts (3.5 ± 0.7 fold increased (Figure 2E). Expression of fut1 mRNA in TNF-α stimulated RA synovial fibroblasts was also significantly elevated compared to that in TNF-α stimulated NL synovial fibroblasts (4.6 ± 0.8-fold increased). To further validate and distinguish cellular fut1 staining in RA ST, we performed dual immunofluorescence staining on RA ST fibroblasts and macrophages. In Figure 2F, the left panel is fut1 staining in RA ST (green). The middle panel is cadherin-11 (fibroblast marker) staining in RA ST (red). The right panel is the merge of the previous two panels. The arrow indicates fut1- and cadherin-11-positive cells respectively (yellow), indicating that fut1 is expressed on fibroblasts in RA ST. In Figure 2G, the left panel is fut1 staining in RA ST (green). The middle panel is CD68 (macrophage marker) staining in RA ST (red). The right panel is the merge of these two panels. The arrows indicate fut1 on CD68-positive cells (yellow), indicating that fut1 is expressed on macrophages in RA ST. The blue background is DAPI staining. IgG control staining was performed and showed no fluorescence staining.

### Blocking fut1 expression in RA synovial fibroblasts reduces EC tube formation

**Figure 3:**
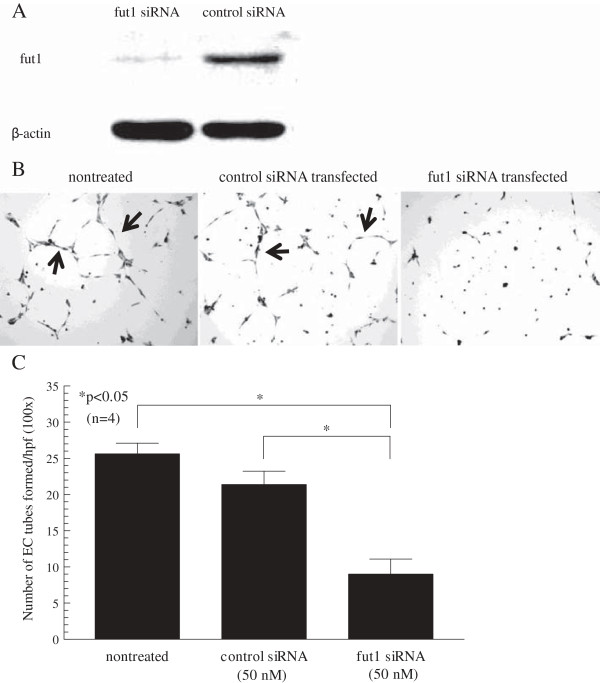
**Fucosyltransferase 1 (fut1) expression was decreased using siRNA against fut1 in rheumatoid arthritis (RA) synovial fibroblasts.** Cells were stimulated with TNF-α (25 ng/ml) for 24 hours. **(A)** Fut1 expression in fut1 siRNA-treated RA synovial fibroblasts and control siRNA-treated RA synovial fibroblasts. **(B)** RA synovial fibroblasts were first transfected with control or fut1 siRNA, and were grown on the top inserts. Human dermal microvascular endothelial cells (HMVECs) were plated in the bottom of the wells of the transwell system for 24 hours. HMVECs were plated on Matrigel with nontreated, control siRNA-transfected or fut1 siRNA-transfected RA synovial fibroblast-conditioned medium for 6 hours. A representative photograph of HMVECs from co-culture with nontreated, control or fut1 siRNA-transfected RA synovial fibroblasts is shown. Arrows indicate tubes. **(C)** HMVECs incubated with fut1 siRNA-transfected RA synovial fibroblast conditioned media formed significantly fewer tubes on Matrigel compared with control siRNA-treated RA synovial fibroblasts or nontreated RA synovial fibroblasts. Means are presented with standard error of the mean. *P <0.05 was significant (n = number of RA patient synovial fibroblasts).

To determine the function of fut1 in RA synovial fibroblasts, we used siRNA directed against fut1. The transfection efficiency in RA synovial fibroblasts was 88 ± 2% (n = 4 patients), and the percent knockdown of fut1 mRNA was 73 ± 1% (n = 4 patients). The specific knockdown of fut1 was confirmed by western blotting, and fut1 protein levels were decreased (Figure 3A). To examine the role of fut1 in angiogenesis with respect to RA, we co-cultured HMVECs with nontreated, control siRNA or fut1 siRNA-transfected RA synovial fibroblasts in an *in vitro* chamber system. HMVECs harvested from the fut1 siRNA-transfected RA synovial fibroblast co-culture system had decreased EC tube formation compared with HMVECs harvested from the control siRNA or the nontreated RA synovial fibroblast co-culture system (number of EC tubes formed per high power field ± SEM; 4 ± 2, 23 ± 1 and 27 ± 1, respectively; *P* < 0.05, Figure 3B and C).

### Blocking fut1 expression in RA synovial fibroblasts reduces expression of proangiogenic mediators

**Figure 4:**
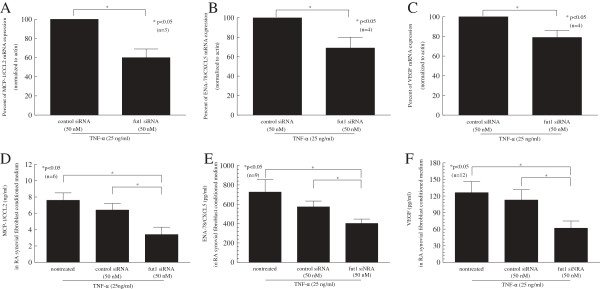
**Fucosyltransferase 1 (Fut1) siRNA inhibited pro-angiogenic mediator production in TNF-α-stimulated rheumatoid arthritis (RA) synovial fibroblasts. (A)** Monocyte chemoattract protein 1 (MCP-1/CCL2) mRNA expression in fut1 siRNA-treated RA synovial fibroblasts was 60 ± 9% (mean ± standard error (SE)) of control siRNA-transfected RA synovial fibroblasts, showing a 40% reduction in MCP-1/CCL2 mRNA expression in fut1 siRNA-transfected cells. **(B)** Epithelial-derived neutrophil-activating peptide 78 (ENA-78/CXCL5) mRNA expression in fut1 siRNA-treated RA synovial fibroblasts was 69 ± 11% (mean ± SE) of control siRNA transfected RA synovial fibroblasts, showing a 31% reduction in ENA-78/CXCL5 mRNA expression fut1 siRNA-transfected cells. **(C)** Vascular endothelial growth factor (VEGF) mRNA expression in fut1 siRNA-treated RA synovial fibroblasts was 79 ± 7% (mean ± SE) of control siRNA-transfected RA synovial fibroblasts, showing a 21% reduction in VEGF mRNA expression fut1 siRNA-transfected cells. **(D)** Fut1 siRNA inhibited production of MCP-1/CCL2. **(E)** Fut1 siRNA inhibited production of ENA-78/CXCL5. **(F)** Fut1 siRNA inhibited production of VEGF. Means are presented with SE. *P <0.05 was significant (n = number of RA patient synovial fibroblasts).

We found that mRNA expression of MCP-1/CCL2, ENA-78/CXCL5 and VEGF in TNF-α-stimulated fut1 siRNA-transfected RA synovial fibroblasts was significantly decreased compared to control siRNA-transfected RA synovial fibroblasts (Figure 4A, B and C). Hence, we measured secretion of MCP-1/CCL2, ENA-78/CXCL5 and VEGF in fut1 or control siRNA-transfected and nontreated RA synovial fibroblast-conditioned medium. The secretion of MCP-1/CCL2, ENA-78/CXCL5, and VEGF in TNF-α-stimulated fut1 siRNA-transfected RA synovial fibroblasts was significantly decreased compared with control siRNA-transfected or nontreated RA synovial fibroblasts (Figure 4D, E and F).

### Fut1 siRNA inhibits THP-1 cell adhesion to RA synovial fibroblasts

**Figure 5:**
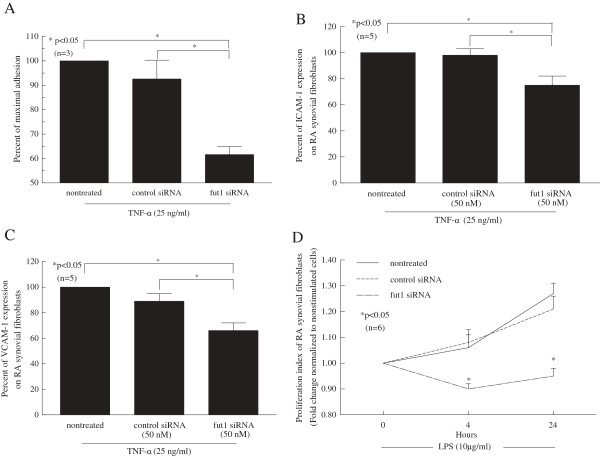
**Fucosyltransferase 1 (fut1) mediates adhesion of THP-1 cells to rheumatoid arthritis (RA) synovial fibroblasts and mediates their proliferation.** The percent adhesion was defined as the number of adherent cells on synovial tissue (ST) sections divided by the number of adherent cells on control sections. **(A)** Adhesion of THP-1 cells to TNF-α-stimulated fut1 siRNA-transfected RA synovial fibroblasts (62 ± 8% of maximal adhesion) was significantly decreased compared with adhesion of THP-1 cells to TNF-α-stimulated control siRNA-transfected (93 ± 8% of maximal adhesion) or nontreated RA synovial fibroblasts. **(B)** Intercellular adhesion molecule 1 (ICAM-1) expression on TNF-α-stimulated fut1 siRNA-transfected RA synovial fibroblasts was significantly decreased compared to TNF-α-stimulated control siRNA or nontreated RA synovial fibroblasts. **(C)** Vascular cell adhesion molecule 1 (VCAM-1) expression on TNF-α-stimulated fut1 siRNA-transfected RA synovial fibroblasts was significantly decreased compared to TNF-α-stimulated control siRNA or nontreated RA synovial fibroblasts. **(D)** Fut1 siRNA-transfected lipolysaccharide (LPS)-stimulated RA synovial fibroblasts showed reduced proliferation at 4 and 24 hours compared to LPS-stimulated control siRNA-transfected or nontreated RA synovial fibroblasts. The results are shown as fold change in optical density of fut1- or control siRNA-transfected RA synovial fibroblasts normalized to nontreated RA synovial fibroblasts. Means are presented with standard error. *P <0.05 was significant. (n = number of RA patient synovial fibroblast cultures).

To determine if fut1 mediates leukocyte adhesion to RA synovial fibroblasts, we performed *in vitro* adhesion assays. We found that adhesion of THP-1 cells to fut1 siRNA-transfected RA synovial fibroblasts in response to TNF-α was significantly decreased compared with that to control siRNA transfected or nontreated RA synovial fibroblasts (Figure 5A). In addition, we performed a cell-surface ELISA to determine if cell adhesion molecules were decreased on the cell surface of fut1 siRNA-transfected RA synovial fibroblasts. We found that ICAM-1 and VCAM-1 on TNF-α-stimulated fut1 siRNA-transfected RA synovial fibroblasts were decreased compared to control siRNA-transfected or nontreated RA synovial fibroblasts (Figure 5B and C). These results confirm that fut1 inhibition regulates TNF-α-induced fibroblast adhesion and adhesion molecule expression.

### Fut1 siRNA inhibits RA synovial fibroblast proliferation

The effect of fut1 on RA synovial fibroblast proliferation was examined. We found that RA synovial fibroblasts transfected with fut1 siRNA showed less proliferation in response to LPS at 4 and 24 hours (Figure 5D).] As shown in Figure 5D, fut1 siRNA-transfected LPS-stimulated RA synovial fibroblast cultures displayed significantly less proliferation at 4 and 24 hours compared to LPS-stimulated control siRNA-transfected or nontreated RA synovial fibroblasts. These results show that fut1 inhibition decreases fibroblast proliferation in response to LPS stimulation.

### Fut1 siRNA inhibits phosphorylated JNK signaling in RA synovial fibroblasts

**Figure 6:**
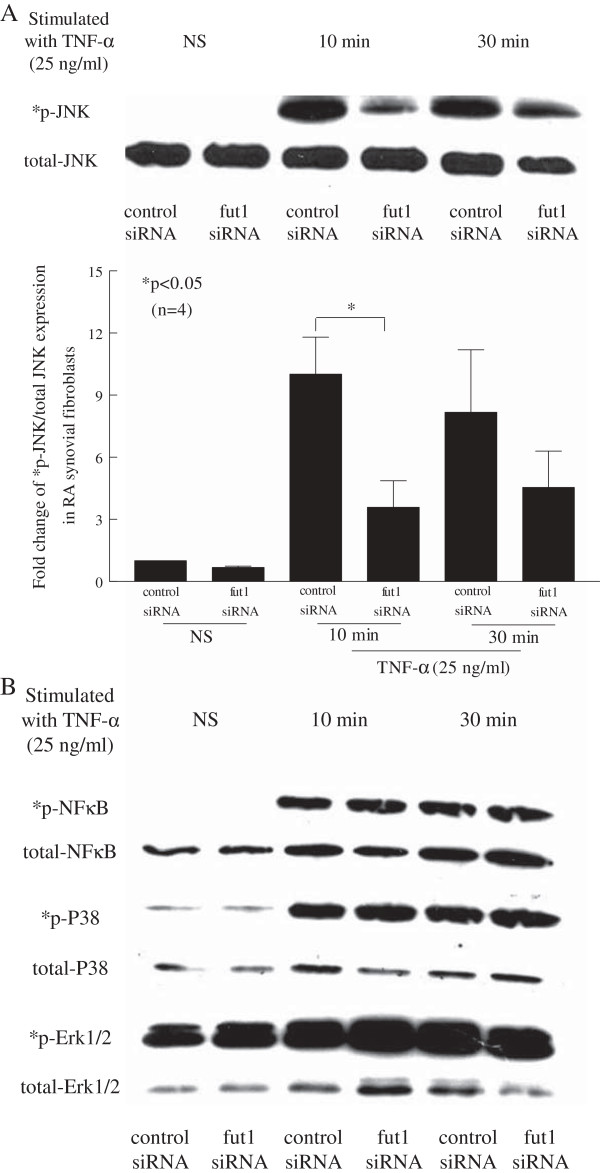
**Fucosyltransferase 1 (fut1) siRNA inhibits phosphorylated JNK signaling in rheumatoid arthritis (RA) synovial fibroblasts. (A)** Western blots were performed to determine whether TNF-α stimulates the phosphorylation of JNK. Phosphorylation of JNK signaling in TNF-α-stimulated fut1 siRNA-transfected RA synovial fibroblasts was significantly decreased at 10 minutes compared to control siRNA-transfected RA synovial fibroblasts. **(B)** Fut1 siRNA does not inhibit phosphorylation of NFκB, P38, and Erk1/2 in RA synovial fibroblasts. Means are presented with standard errror. *P <0.05 was significant; *p indicates phosphorylated signaling proteins (n = number of RA patient synovial fibroblasts).

To determine RA synovial fibroblast fut1-associated signaling mechanisms, western blot was performed using fut1 or control siRNA-transfected RA synovial fibroblasts stimulated with TNF-α (25 ng/ml) for 10 and 30 minutes. We found that phosphorylated JNK signaling in TNF-α-stimulated fut1 siRNA-transfected RA synovial fibroblasts was significantly decreased at 10 minutes compared to control siRNA-transfected cells (Figure 6A). However, phosphorylated NFκB, P38, and Erk1/2 signaling were not different between fut1- and control siRNA-transfected RA synovial fibroblasts (Figure 6B). These results indicate that early phosphorylation of JNK is important for fut1 signaling in RA synovial fibroblasts.

## Discussion

Posttranslational modifications, such as glycosylation, citrullination or NH_2_-terminal truncation of natural cytokines, change their biological activity [30,31]. Nabeshima *et al*. reported that the cytokine glycosylation on receptor binding changed biological activity [32]. These reports indicate that glycosylated cytokines may contribute to disease pathogenesis. Over half of known proteins are modified by covalently bound glycans, which are important for physiological processes including protein folding, degradation, signaling, and immune function [33]. The complexity of the glycoproteome is thought to be several orders of magnitude greater than the proteonome [33]. Human ABO blood group antigens and Lewis systems are oligosaccharides synthesized by sequential actions of futs and these antigens are important in blood typing [34]. The α(1,2)-fucosyltransferases fut1 and fut2 are the enzymes responsible for catalyzing an α(1,2)-linkage of fucose to terminal beta galactosidase [35]. H and Lewis antigens are expressed most abundantly in endodermal epithelial cells, where the majority of human cancers arise [36].

We hypothesized that fut1 in RA is overexpressed, and mediates angiogenesis, cell adhesion, and fibroblast proliferation. Indeed, we found that α(1,2)-linked fucosylated proteins were overexpressed in RA. Fucosylation is one of the most common modifications involving oligosaccharides on glycoproteins, and their structures are involved in a variety of biological processes in eukaryotic organisms, angiogenesis, fertilization, cell adhesion, inflammation, and tumor metastasis [37]. We and others have previously reported that sialyl Lewis^x^, synthesized by α(1,3)-fucosyltransferases, is involved in angiogenesis [21]. In addition, we reported that soluble H and Lewis^y^ antigens, both synthesized by fut1, are potent mediators of cell adhesion, angiogenesis, and monocyte recruitment [22,38,39]. Our study clearly demonstrates that α(1,2)-linked fucosylated proteins are more highly expressed on RA synovial fibroblasts than on NL synovial fibroblasts. Przybysz *et al*. showed that the expression of α(1,6)-linked fucose in synovial fibronectins was related to RA disease activity [40]. Kratz *et al*. showed that the proportions of fucosyl determinants of intact synovial IgA and IgG were lower in the early RA group compared to the advanced RA group [41], suggesting that fucosylated antibodies may be important in chronic RA pathogenesis. These findings suggest that α(1,2)-linked fucosylation has an important role in RA.

We next focused on fut1 expression and function in RA tissues. Fut1 is overexpressed in some cancers such as colon and pancreas. Fut1 mRNA in cancer tissues was elevated compared to normal tissues [17,18]. Thus far, there have been no reports of fut1 in RA. We examined a potential relationship between lining cells and lining thickness score, however there was not a correlation between them. Nonetheless, we and others have shown that angiogenesis is important in the growth and proliferation of the RA ST pannus, and in the ingress of leukocytes, and that cytokines play a key role in this process [9,20,42]. We also found that HMVECs from a co-culture system with fut1 siRNA-treated RA synovial fibroblasts had decreased HMVEC tube formation compared with HMVECs from a similar co-culture system with control siRNA-treated or nontreated RA synovial fibroblasts. This is in agreement with Mathieu *et al*. who showed that fut1-deficient hepatocarcinoma cells had reduced angiogenic responses [43].

After defining the activity of fut1 using HMVEC tube formation assays with RA synovial fibroblasts, we assessed the expression of pro-angiogenic mediators from fut1 siRNA-transfected RA synovial fibroblasts. We found that MCP-1/CCL2, ENA-78/CXCL5, and VEGF mRNA in TNF-α-stimulated fut1 siRNA-transfected RA synovial fibroblasts were decreased compared to control siRNA-transfected RA synovial fibroblasts. We also found that secretion of MCP-1/CCL2, ENA-78/CXCL5, and VEGF in TNF-α-stimulated fut1 siRNA-transfected RA synovial fibroblasts was decreased compared to control siRNA-transfected RA synovial fibroblasts. These findings suggest that fut1 expressed in synovial fibroblasts is important in RA angiogenesis by contributing to the production of pro-angiogenic mediators.

We next examined the role of fut1 in leukocyte adhesion. Leukocyte retention in the synovium is an active process mediated in part by cellular adhesion molecules [44]. We found that adhesion of myeloid THP-1 cells to fut1 siRNA-transfected RA synovial fibroblasts was significantly decreased compared with control or nontreated RA synovial fibroblasts. These findings are consistent with Palumberi *et al*. who showed that adhesion of human epidermoid carcinoma cells to fut1 and fut2 siRNA-transfected ECs was decreased compared with control siRNA-transfected ECs [45]. On the other hand, Kwiatkowski *et al*. reported that EC surface expression of terminally sialylated structures by high-level fut1 activity reduced monocyte adherence and activation [46]. However, this group did not examine which adhesion molecules were differentially expressed during fut1 inhibition in ECs. In addition, the former group used bovine post-capillary venular ECs that bind to epidermoid carcinoma cells, while the other group used porcine EC monolayers to examine monocyte adhesion. Perhaps the different type of ECs, the use of epidermoid carcinoma cells, along with the different cell isolation methods could account for the differences in cellular adhesion. It could also be that overexpression of futs may have limits, and that highly elevated levels of fut activity may actually inhibit monocyte and EC interactions, at least in *in vitro* systems. Nonetheless, we found that cell-surface adhesion molecules such as ICAM-1 or VCAM-1 on fut1 siRNA-transfected RA synovial fibroblasts were decreased compared to control fibroblasts. These findings indicate that fut1 in RA synovial fibroblasts is not only important for cell adhesion, but indicate that these interactions may also lead to activation of inflammatory cells and perpetuation of inflammation in RA synovium.

RA synovial fibroblasts proliferate and invade cartilage [47]. We found that fut1 siRNA inhibits cell proliferation of LPS-stimulated RA synovial fibroblasts. Interestingly, Palumberi *et al*. also reported that fut1 and fut2 siRNA-treated human epidermoid carcinoma cells have reduced cell proliferation when transfected with fut1 and fut2 siRNA [45]. In agreement with Palumberi, we found that fut1 facilitates fibroblast proliferation, indicating that α(1,2)-linked fucosylation by fut1 may contribute to fibroblast overgrowth in the RA pannus.

Finally, we found that fut1 siRNA inhibited phosphorylated JNK signaling in RA synovial fibroblasts. On the other hand, fut1 siRNA did not inhibit phosphorylation of NFκB, P38, and Erk1/2 signaling in RA synovial fibroblasts. Overall, our results demonstrate that JNK plays key roles in mediating angiogenesis, cell adhesion and RA synovial fibroblast proliferation through fut1.

## Conclusion

Our study determined that α(1,2)-linked fucosylation of fibroblasts is important in RA. We have shown that α(1,2)-linked fucosylated proteins are highly expressed in RA synovial fibroblasts compared to NL synovial fibroblasts. We also have demonstrated that fut1 is expressed on RA synovial lining cells and macrophages. Most importantly, we have shown that fut1 in RA synovial fibroblasts contributes to angiogenesis, cell adhesion, and cell proliferation. We propose that fut1 plays roles in mediating arthritis by this multistep process. Taken together, these results demonstrate the importance of α(1,2)-linked fucosylation by fut1 in RA and suggest that targeting fut1 may be important in combating RA.

## Abbreviations

2′FL-BSA: 2′fucosyllactose-bovine serum albumin; DAB: chromogen 3,3′-diaminobenzidine; DAPI: 4′,6-diamidino-2-phenylindole; ECs: endothelial cells; ELLA: enzyme-linked lectin assay; ENA-78/CXCL5: epithelial-derived neutrophil-activating peptide 78; FBS: fetal bovine serum; FITC: fluorescein isothiocyanate; fut1: fucosyltransferase 1; futs: fucosyltransferases; H-2 g: 2-fucosyllactose; HMVEC: human dermal microvascular endothelial cell; ICAM-1: intercellular adhesion molecule 1; IL-6: interleukin-6; Ley/H: Lewis^y^-6/H/5-2; LPS: lipopolysaccharide; MCP-1/CCL2: monocyte chemoattractant protein 1; NL: normal; OA: osteoarthritis; PBS: phosphate-buffered saline; qPCR: quantitative polymerase chain reaction; RA: rheumatoid arthritis; RPMI: Roswell Park Memorial Institute; SEM: standard error of the mean; siRNA: small interfering RNA; ST: synovial tissue; TNF-α: tumor necrosis factor-α; UEA-1: Ulex Europeaus Agglutinin 1 lectin; VCAM-1: vascular cell adhesion molecule 1; VEGF: vascular endothelial growth factor.

## Competing interests

The authors declare that they have no competing interests.

## Authors’ contributions

Conception and design by AEK and TI. Acquisition of data by TI, JHR, PT, CMH, (GE) and GKH. Analysis and interpretation of data by TI, JHR, MAA, PLC, (GE) and AEK. Drafting of manuscript by TI, JHR, MAA, PLC, and AEK. All authors read and approved the final manuscript.
